# A Case of Complete Ossification of the Fœtal Head

**Published:** 1857-05

**Authors:** 


### *A Case of Complete Ossification of the Fœtal Head.*
By Prof. Allen.

I was called in consultation by Dr. Haynes of this place, to see Mrs. M——, in protracted labor. There had been vigorous uterine action for twelve hours, the waters had been discharged, the head presenting in the first position. The relative capacities of the head and pelvis seemed properly proportioned, yet there had been no advance of head for many hours. After making thorough exploration, no efficient cause for the tardy expulsion could be arrived at. There could no fontanellas be traced—the sutures were closely adpested, and indeed a miniature *adult* head was presenting. There could be no laping of the bones, no diminution of size from compression, and cramotomy was determined upon. So firm was the osseous matter, that the performator could not be introduced by any safe force. Prof. Hughes, was then requested to see the case, and bring some instrument, by which the bone could be penetrated. By persevering drilling, and the use of a bone saw, the head was, at last, opened, the brain removed, the forceps applied, and the head, after some considerable effort, was brought into inferious passages and delivered. On examination, the sutures were found as firmly united as on the adult—the osseous matter almost flinty. The difficulty was, undoubtedly, in the non-compressibility of the head, as the proportions were such as would under the ordinary state of the fœtal head, have been entirely consistent with a natural and easy delivery.

---

*Perchloride of Iron as an Hæmostatic.*—A correspondent of the *Moniteur des Hopitaux* (No. 24, 1856), states that one of the principal elements of his success in the difficult and dangerous operations M. Maissonneuve is famous for undertaking, is the remarkable use he makes of hæmostatics during their performance. He cites a recent case, occurring in a lad of sixteen, of fungous tumor of the dura mater, the growth of which, after having been temporarily arrested by ligature of the carotid, took on enormous proportions, and was accompanied by exhausting hemorrhages. M. Maissonneuve determined upon its removal, but the tumor bled on the slightest contact, and the patient would not be able to bear the slightest loss of blood. The line of incision extended from the anterior parts of the ear to the summit of the head, and descending along the nose, was carried backwards, and then upwards to the base of the jaw, and its point of departure. A great number of arteries were thus divided, five or six of which, by reason of their anastomotic enlargements, had acquired almost the size of the radial artery. Intelligent assistants immediately compressed them with the finger, but it was impossible to thus continue the dissection without exposing the patient to the danger of death from syncope. M. Maissonneuve therefore applied to each vessel a little pledget of charpie soaked in perchloride of iron, which was allowed to attach itself to the wound. At every stroke of the bistoury or scissors he applied a new plug, so that during the operation the patient scarcely lost a spoonful of blood; and when the tumor had been entirely removed, the entire surface of the wound was found completely dried and tanned, and was at once dressed, without the necessity of the application of a single ligature. The brown eschar which covered the wound was detached about the twentieth day, without giving rise to any hemorrhage; and although the cure can scarcely be expected to prove radical, the patient, for the present, is perfectly well.—*Med. Times and Gazette, Aug.* 16*th*, 1856.

---

*A very large Ovarian Tumor.* By Dr. Geo. D. Gibb.

This tumor is probably the largest ever removed entire from the dead body, its weight being no less than 106 pounds. It was obtained from a young unmarried woman, æt. 31, and, so far as could be ascertained, it had occupied about seven years in its growth. As might be expected from its extreme size, the displacement of the viscera was very remarkable, the stomach and the greater part of the bowels occupying the position of the left lung, which was completely shrunk and carnified, the cervix uteri and vagina being remarkably elongated, etc. The tumor itself was composed of simple fibro-cellular tissue, with some cysts—a large one, containing forty-four pints of homogeneous puriform fluid, and several minute ones, containing a yellowish red, serous-looking fluid,—but none of these cysts appeared to be proliferous—*Ranking's Half-Yearly Abstract.*

